# Unmasking *Prototheca wickerhamii*: A rare case of cutaneous infection and its implications for clinical practice

**DOI:** 10.1016/j.bjid.2025.104525

**Published:** 2025-03-27

**Authors:** Jing Li, Zeyu Huang, Ruzhi Zhang

**Affiliations:** a The Second Affiliated Hospital of Wannan Medical College, Department of Dermatology, Jinghu District, Wuhu, PR China; b The Third Affiliated Hospital of Soochow University, Department of Dermatology, Changzhou, PR China

**Keywords:** Achlorophyllic algae, Infection, Diagnosis, Treatment

## Abstract

*Prototheca*, an opportunistic pathogenic algae widely found in nature, has emerged as a potential public health concern. Most cases occur in immunocompromised individuals, with infections in immunocompetent patients being relatively rare. Due to their non-specific clinical presentation and limited awareness among clinicians, *Prototheca* infections are often misdiagnosed, resulting in delayed treatment. Recent advances in species identification and antifungal susceptibility testing have provided important tools for diagnosis and therapy. Here, we report a case of recurrent facial infection in a 76-year-old immunocompetent man. Skin biopsy revealed an infectious granuloma, and fungal culture identified yeast-like colonies. Fluorescence staining and scanning electron microscopy revealed abundant spores, while metagenomic sequencing confirmed the infection as *Prototheca wickerhamii*. The patient was successfully treated with long-term itraconazole and dipotassium glycyrrhizinate capsules. This case highlights the importance of early and accurate diagnosis in the management of *Prototheca* skin infections and reviews the therapeutic strategies used.

## Introduction

*Protothecosis* is a rare opportunistic infection caused by microorganisms of the genus *Prototheca*, with *Prototheca wickerhamii* being the primary human pathogen. *P. wickerhamii* is a unicellular microorganism, 3–30 μm in diameter, classified in the phylum Chlorophyta and class *Trebouxiophyceae*. It is commonly found in environments such as sewage, soil, raw milk, the surfaces of plants and animals, and even human skin, nails and feces. The organism reproduces asexually via endospores, and infection is likely associated with trauma or exposure to contaminated water.^1^ Although *protothecosis* was historically rare, the incidence of protothecosis has increased significantly over the past decade, paralleling the growth of the immunocompromised population worldwide.^2^ However, treatment guidelines for this infection remain unclear and diagnosis is often delayed or misdiagnosed due to non-specific clinical presentations. In this report, we present a case of facial *P. wickerhamii* infection in an immunocompetent patient. Through detailed pathologic and microbiologic investigations, we review the diagnostic and therapeutic approach in this case to provide clinical insights for practitioners.

## Case report

**Fig. 1:**
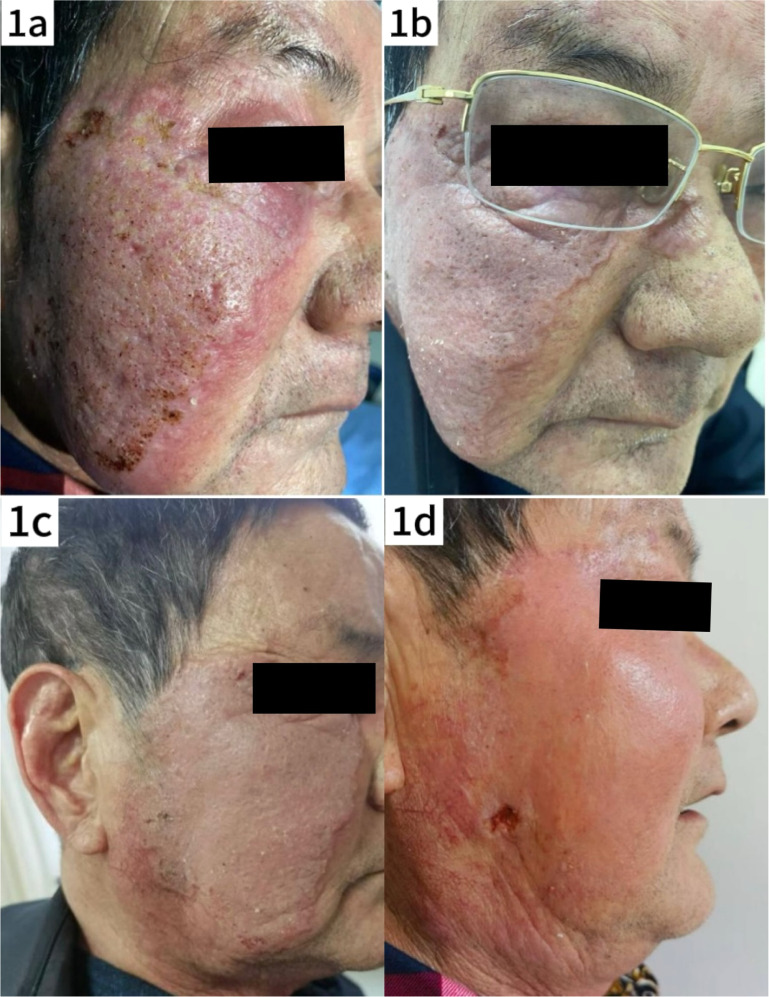
Clinical evolution of the patient's skin lesions. (a) Photograph taken at the patient's initial visit shows red plaques with mild infiltration, well-defined borders, scaling and elevated local skin temperature. (b) Photograph taken before discharge after inpatient treatment, showing significant reduction in facial swelling. (c and d) Photographs taken during follow-up after the patient's self-treatment, showing new areas of mild erosion and worsening of the lesions compared to the previous condition.

The patient, a 76-year-old man, presented with a two-month history of a plaque on the right side of his face, accompanied by mild pruritus. The lesion had well-defined borders, with prominent surrounding erythema and localized telangiectasia, but no significant ulceration (Fig. 1a). The patient reported a history of living outdoors and had self-administered topical corticosteroids, which did not improve the rash; instead, the lesion expanded, and pustules with thin crusting appeared on the surface, although there was no significant exudation. On admission to the hospital, blood tests revealed an elevated C-Reactive Protein (CRP) level of 42.40 mg/L (normal range < 10 mg/L), indicating a possible soft tissue infection. Initial treatment did not include a skin biopsy, and the patient was treated with antibiotics (oral azelastine 2 mg bid and intravenous cefuroxime sodium 1.5 g bid) with a poor response. The regimen was then changed to intravenous amoxicillin-clavulanate 1.2 g bid in combination with intravenous dexamethasone 5 mg qd and oral itraconazole 0.2 g bid. After one week of treatment, the rash showed marked improvement (Fig. 1b), and the patient was discharged on medication.

One week after discharge, the patient discontinued the medication on his own, and approximately one month later, the rash worsened, necessitating readmission for further management. Dermatologic examination revealed significant facial swelling with ulcerated lesions, while the plaque maintained well-defined borders and localized skin warmth was noted (Fig. 1c). Blood tests revealed a white blood cell count of 7.29 × 10^9/L, neutrophil percentage of 86.0 %, Alanine Aminotransferase (ALT) at 88 U/L, Aspartate Aminotransferase (AST) of 36 U/L, and no other significant abnormalities. With the consent of the patient and his family, a skin biopsy was performed and tissue samples from the lesions were sent for fungal and bacterial culture and metagenomic sequencing.

### Pathology report

**Fig. 2:**
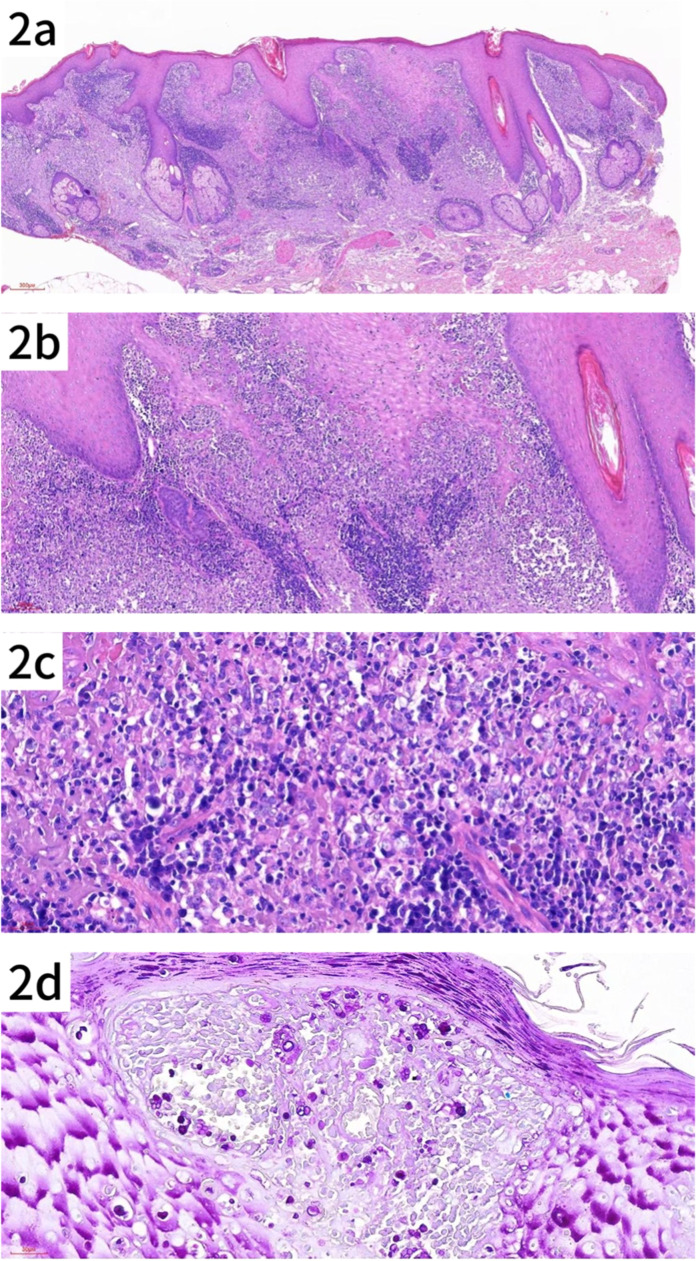
Histologic features of the patient's skin lesion. (a) Pseudoepitheliomatous hyperplasia with dense infiltration of lymphocytes, histiocytes and plasma cells in the superficial and mid-dermis (HE × 40). (b) Higher magnification showing the inflammatory infiltrate in the dermis (original magnification × 100). (c) Close-up of the infiltrate, highlighting individual cells within the lesion (original magnification × 200). (d) Spores of various sizes, some with a mulberry or soccer ball appearance, observed in both superficial and deeper layers of the epidermis (PAS × 200).

Pathologic findings included hyperkeratosis with parakeratosis, and pseudoepitheliomatous hyperplasia of the epidermis. The superficial and mid-dermis showed a dense infiltration of inflammatory cells, including lymphocytes, histiocytes, neutrophils and plasma cells, some of which infiltrated the epidermis. Special staining showed PAS-positive (+) and acid-fast negative (-) results, consistent with an infectious granuloma (Fig. 2a–d).

### Microbiological examination

**Fig. 3:**
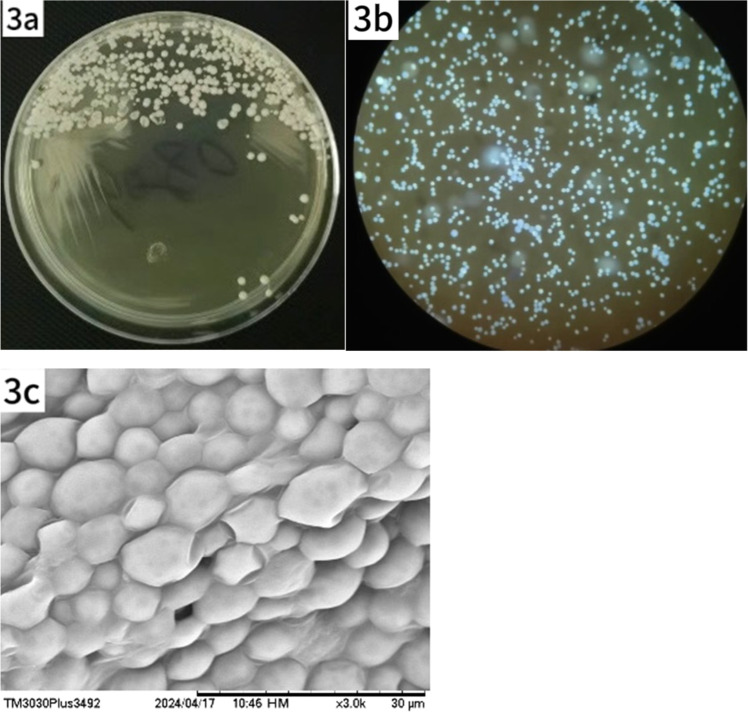
Growth and morphological characteristics of Prototheca wickerhamii under light and SEM. (a) Creamy, yeast-like colonies observed on SDA after 4 days of incubation at 30 °C. (b) Microscopic examination reveals numerous spores, some with visible septation. (c) SEM shows spherical clusters of Prototheca wickerhamii cells.

No microbial growth was observed after 2days of culture; however, yeast-like colonies appeared on day 4 (Fig. 3a). Fluorescence staining of the colonies revealed abundant spores (Fig. 3b). Moist, gray-white, creamy colonies were visible to the naked eye on Sabouraud Dextrose Agar (SDA) at 30 °C. Microscopic examination of a 10 % Potassium Hydroxide (KOH) preparation revealed numerous round and oval spores that were transparent, thick-walled, and lacked hyphae or budding. Intracellular endospores resembling a mulberry-like structure were also observed. A colony sample was examined by Scanning Electron Microscopy (SEM) (Fig. 3c).

### Pathogen sequencing of the pathogen

**Fig. 4:**
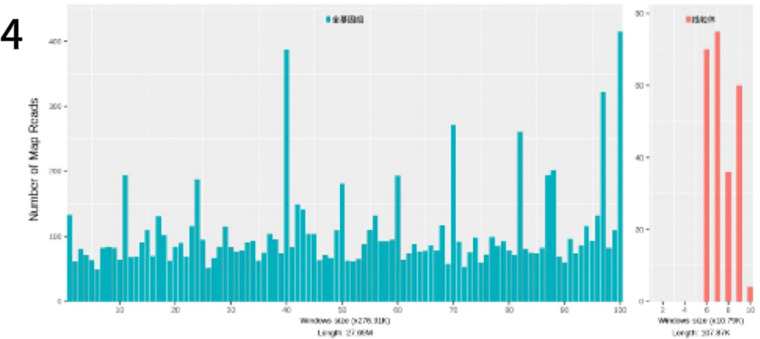
Genomic and mitochondrial coverage of Prototheca wickerhamii identified by metagenomic sequencing. The figure shows the genomic coverage of *Prototheca wickerhamii*, with the whole genome on the left side and the mitochondrial genome on the right.

To identify the pathogenic microorganism, a small skin pathology tissue sample was collected with the informed consent of the patient and her family and sent to the Translational Medicine Center laboratory of our hospital for further analysis. Pathogen nucleic acid sequencing was performed using probe-based capture high-throughput sequencing (MetaCAP™)^3^ and confirmed Prototheca wickerhamii as the causative agent (Fig. 4). The metagenomic sequencing data revealed 3423 sequences of this microorganism with a relative abundance of 99.3 % and a confidence level of 99 %.

### Treatment course

**Fig. 5:**
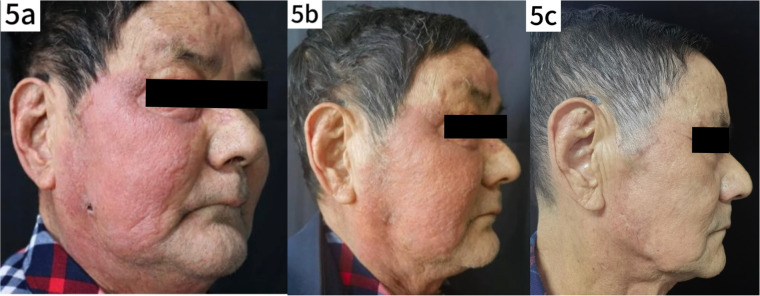
(a) Photograph taken after adjustment of treatment regimen following confirmed diagnosis of *protothecosis*. (b) Photograph showing improvement one week after discharge. (c) Follow-up photograph after five months of treatment, documenting complete resolution of lesions.

Following the confirmed diagnosis of *Prototheca wickerhamii* infection, the treatment regimen was adjusted accordingly. To alleviate the symptoms of pruritus and edema (Fig. 1d), intravenous dexamethasone 5 mg qd was administered along with oral itraconazole 0.1 g bid for antifungal treatment. After treatment, the facial swelling decreased significantly, although the local skin temperature remained elevated (Fig. 5a). Blood tests showed a neutrophil count of 86.8 %, a lymphocyte count of 7.4 %, and a decrease in C-reactive protein to 0.80 mg/L. Liver function tests were elevated with Alanine Aminotransferase (ALT) at 121 U/L and Aspartate Aminotransferase (AST) at 47 U/L. Given the liver function abnormalities, the patient was discharged with continued oral itraconazole 0.1 g bid, supplemented with dipotassium glycyrrhizinate capsules 100 mg tid to support liver function. Steroid therapy was gradually tapered. At the one-week follow-up after discharge, the patient's skin lesions showed marked improvement (Fig. 5b). After five months of continued treatment, the lesions had completely resolved, and the patient reported high satisfaction (Fig. 5c).

## Discussion

Although *Prototheca wickerhamii* infections are relatively rare, advances in microbial culture techniques and molecular biology have led to an increased detection rate. To date, approximately 200 cases of *protothecosis* have been reported worldwide, with approximately 30 cases documented in China.^4^^,^^5^ The primary clinical manifestations include cutaneous infections, synovitis, fibrositis and disseminated infections. While cutaneous *protothecosis* generally has a favorable prognosis, with a mortality rate of only 1 %, disseminated infections carry a significantly higher mortality rate of 56 %. Although the pathogenic mechanisms of *Prototheca* remain incompletely understood, immunocompromised individuals are at highest risk for developing disseminated *protothecosis* with a mortality rate much higher than that of localized skin infections.^6^^,^^7^ In this case, the patient may have experienced a decline in local immunity due to prolonged use of topical corticosteroids, making them susceptible to *Prototheca* infection while working outdoors.

The cutaneous manifestations of *protothecosis* are often polymorphic, with the face and extremities being the primary sites of infection. Due to a lack of awareness of prototheca among clinicians, the early symptoms of cutaneous infections are often misdiagnosed or lead to delayed treatment. Common presentations include erythema, papules and plaques, which may be associated with superficial ulcers, necrotic crusts, pustules or bullae. Lesions sometimes resemble eczema but typically have an asymmetric distribution. Facial lesions in particular must be differentiated from erysipelas. Accurate identification of the *Prototheca* is critical to patient prognosis. While traditional microbiological methods can initially differentiate based on morphology, this process can be equivocal. In 2014, Kano et al. successfully diagnosed a case of *protothecosis* using histopathological methods, providing a viable approach to differentiate *Prototheca* from Candida.^8^ Furthermore, studies have shown that the ITS region of pathogenic *Prototheca* has species-specific features, with the ITS region length of small *Prototheca* being 2.6 kb, which serves as an important basis for species differentiation.^9^^,^^10^

In recent years, sequencing technologies have been widely used for microbial identification. MetaCAP™ (Metagenomic Capture) is a novel high-throughput sequencing technique based on metagenomic Next-Generation Sequencing (mNGS).^11^ This method uses millions of custom-designed specific probes that hybridize to microbial nucleic acids in the sample, thereby enriching and capturing sequences from the target region. It provides a cost-effective, rapid, and highly sensitive targeted alternative to whole metagenomic sequencing. Compared to traditional culture methods, next-generation sequencing has demonstrated higher pathogen detection rates in infectious diseases affecting the bones, joints, and skin.^12^^,^^13^ As a culture-independent detection approach, MetaCAP is applicable to various clinically suspected infection specimens, particularly in cases where traditional cultures yield negative results, allowing infection assessment in conjunction with clinical presentation.^14^ In this case, we also confirmed the infecting pathogen as *Prototheca wickerhamii* by metagenomic sequencing.

Currently, the Clinical and Laboratory Standards Institute (CLSI) in the United States has not established standards for in vitro antifungal susceptibility testing for *Prototheca*. Some laboratories rely on the yeast susceptibility testing guidelines (CLSI M27-A3) for evaluation.

In vitro susceptibility testing results indicate that *Prototheca* is most susceptible to amphotericin B, followed by voriconazole.^15^ No strains resistant to amphotericin B have been identified in the reported cases in China.^5^ However, given the patient's mild hepatic and renal dysfunction, the use of amphotericin B ‒ known for its hepatotoxicity and nephrotoxicity ‒ was considered inappropriate. As a result, itraconazole was administered along with hepatoprotective therapy, resulting in a prolonged treatment duration. The literature indicates that *P. wickerhamii* often shows resistance to 5-fluorocytosine and fluconazole (Diflucan), while itraconazole and voriconazole show superior efficacy compared to other antifungal agents.

In addition, Kwiecinski et al. reported that *Prototheca* can form biofilms containing cells at different growth stages, DNA and polysaccharides, and that their formation is regulated by host plasma.^16^ The development of biofilms can induce resistance in *Prototheca* and contribute to chronic, refractory infections. Research has also shown that successful treatment depends not only on antifungal agents, but also on thorough debridement and irrigation of the infected area to prevent further spread of the infection.^17^^,^^18^ To reduce the risks associated with *Prototheca wickerhamii* infections, including serious complications and mortality, more research is needed to develop new treatment strategies and clinical guidelines.

In conclusion, the threat of *Prototheca* infections to human health is gradually increasing. Clinicians should deepen their understanding of *Prototheca* and its microscopic morphology in microbial examination and histopathologic diagnosis, and remain vigilant for potential infections to avoid misdiagnosis and treatment delays. Through this case report, we aim to increase clinicians' awareness of *Prototheca* infections and emphasize the importance of timely and accurate differential diagnosis. In particular, it is critical to recognize the risk of localized infections progressing to disseminated infections to prevent potentially fatal outcomes.

## Consent to participate

Written informed consent was obtained from the patient for publication of case details along with images or videos.

## Institutional approval

Approved.

## Conflicts of interest

The authors declare no conflicts of interest.
